# Emerging role of human endogenous retroviruses in mothers with history of perinatal depression and risk of neurodevelopmental disorders in offspring: Results from a pilot study

**DOI:** 10.1371/journal.pone.0351308

**Published:** 2026-07-21

**Authors:** Chiara Cipriani, Martina Siracusano, Martina Giudice, Vita Petrone, Assia Riccioni, Elisa Carloni, Ilaria Adulti, Irene Sferra, Martino Tony Miele, Marialaura Ferrara, Enrico Garaci, Claudia Matteucci, Paola Sinibaldi-Vallebona, Cinzia Niolu, Luigi Mazzone, Emanuela Balestrieri

**Affiliations:** 1 Department of Experimental Medicine, University of Rome Tor Vergata, Rome, Italy; 2 Department of Biomedicine and Prevention, University of Rome Tor Vergata, Rome, Italy; 3 Child Neurology and Psychiatry Unit, Department of Wellbeing of Mental and Neurological, Dental and Sensory Organ Health, Policlinico Tor Vergata Foundation Hospital, Rome, Italy; 4 Psychiatry and Clinical Psychology Unit, Department of Wellbeing of Mental and Neurological, Dental and Sensory Organ Health, Policlinico Tor Vergata Foundation Hospital, Rome, Italy; 5 Systems Medicine Department, University of Rome Tor Vergata, Rome, Italy; 6 IRCCS San Raffaele Pisana, Rome, Italy; California Baptist University, UNITED STATES OF AMERICA

## Abstract

Perinatal depression (PD) actually affects 10−15% of pregnant women and represents one of the most debated topics as potentially implicated in offspring neurodevelopmental disorders etiology, particularly Autism Spectrum Disorder (ASD). Scientific evidence supports the role of Human Endogenous Retroviruses (HERVs) in ASD, as a link among environmental stimuli, epigenetic remodeling and biological processes. The aim of the present study was to characterize the expression profile of different HERVs and selected cytokines in peripheral blood mononuclear cells from women who have experienced PD in comparison to women without history of PD and their respective children stratified according to ASD diagnosis, by RT Real-Time PCR. We showed that ASD children and their PD mothers share abnormal expression of pHERV-W, syncytin-2 and IL-6 likely influenced by the common environment, maternal status, genetic predisposition, or postnatal factors. Of note, mothers with a history of PD were also evaluated at the time of blood sampling using the Hamilton Depression Rating Scale, and a positive correlation with pHERV-W levels was observed. Together with previous results in preclinical models and human studies, our results support the role of HERVs in autism as a contributing factor in creating an adverse environment for normal neurodevelopment, strengthening the view of a mother-child association in the context of autism. PD being associated with the altered activity of HERVs could be considered to be an additional risk factor in the pathogenesis of autism.

## 1. Introduction

Scientific evidence has pointed out the role of prenatal immune environment in the pathogenesis of neurodevelopmental disorders (NDDs), highlighting the close inter-connection between maternal immune activation (MIA) and developmental trajectories in offspring. The prenatal immune environment seems to be influenced by several maternal issues including not only physical (infections, chronic immune diseases, obesity) but also psychological (stress, depression, anxiety) conditions [1,2]. However, until now, no clear relationship has been demonstrated between psychological maternal factors, influencing prenatal environment, and derailed child developmental trajectories.

Perinatal Depression (PD), defined as depression arising during the peripartum period (major depressive disorder with peripartum onset), which includes pregnancy (ante-natal) or the first 4 weeks of postpartum (post-natal) [3] actually affects 10−15% of women [4], and represents one of the most debated topics as potentially implicated in offspring NDDs etiology. Among neurodevelopmental conditions, Autism Spectrum Disorder (ASD) has been examined in this perspective; however, to date, findings remain inconsistent and no clear causal link between maternal PD and ASD risk has been established [5–8]. ASD is a neurodevelopmental disorder characterized by a socio-relational impairment, restricted interests and repetitive behavior [3]. Its etiology is still unclear, although increased ASD risk has been associated with exposure to environmental insults during sensitive prenatal windows [1,9]. In this context, several studies have investigated Human Endogenous Retroviruses (HERVs) as potential link between environmental stimuli, epigenetic remodeling and biological processes implicated in ASD [10,11]. HERVs are ancestral retroviral elements, integrated into germline cells [12,13], and now account for approximately 8% of the human genome [14–15]. HERVs have been implicated as drivers of certain physiological processes, particularly during embryogenesis, pregnancy and immune response [16–20]. Beyond these roles, aberrant reactivation of specific HERVs has been implicated in the pathogenesis of complex conditions, including cancer, autoimmune diseases, and neuropsychiatric and neurodevelopmental disorders [21,22]. In the context of autism, our previous works showed that autistic children and their mothers share abnormal expression of selected HERV families [HERV-H and human endogenous MER34 (medium-reiteration-frequency-family-34) ORF (HEMO)] together with cytokines, including Tumour necrosis factor (TNF)-α, Interferon (IFN)-γ, and Interleukin (IL)-10 [23,24]. Of note, peripheral blood mononuclear cells (PBMCs) of ASD children and their mothers also share responsiveness to microenvironmental stimuli, with *in vitro* exposure to interleukin-2/Phytohaemagglutinin or valproic acid (VPA) inducing the expression of several HERVs and cytokines, while efavirenz inhibiting them [25]. Altered HERV-W expression has been reported in patients with major depression [26], and recent evidence suggests that Syncytin-1 (Syn-1) may modulate stress-related behavioral dysregulation, including suicidal behavior [27].

Based on these considerations, the aim of this pilot study was to assess whether the expression of selected HERVs, syncytins and cytokines might represent a shared biological signature between mothers with a history of PD and their children. This investigation has an exploratory nature since it was conducted using a retrospective cross-sectional design, with biological samples collected some years after pregnancy.

## 2. Materials and methods

### 2.1. Study population

For the purpose of this study, we included two groups of women and their offspring: women who suffered from perinatal depression (PD) and women not affected by mood disorders during pregnancy (NPD) and their children with Autism Spectrum Disorder (ASD) or typical development (TD). The two groups of women and respective children have been enrolled in the context of different previous studies [5,24]. For the first group, enrollment started on the 16^th^ July 2020 and ended on the 16^th^ July 2021; for the second one, enrollment started on 10^th^ June 2013 and ended on 30^th^ November 2013. The PD group was constituted by women and their children, involved in a clinical study focused on perinatal depression and child development, screened during pregnancy (1st or 2nd trimester) for the presence of PD in the context of the SOS MOOD project [5]. This project is a screening program for the early detection of maternal perinatal depression and offspring’s cognitive and behavioral development. Otherwise, the NPD group was constituted by a subgroup of mothers and their offspring, included in projects focused on ASD and HERVs, without history of PD. For more details, see methods sections of the cited study [24]. Demographic characteristics of mothers and children included in the study are reported in Tables 1 and 2.

**Table 1:** Demographic and clinical characteristics of mothers belonging to PD and NPD groups.

Group	N Mothers	Age (years)	Months From Delivery to Sampling	EPDS Score During Pregnancy	HAM-D Score at Follow-up
**PD–ASD**	8	41 ± 5.3	47 months	≥12 (prospective EPDS screening)	10.5 ± 6.4
**PD–TD**	8	41 ± 5.3	47 months	≥12 (prospective EPDS screening)	10.5 ± 6.4
**NPD–ASD**	16	39.1 ± 3.6	68 months	Retrospective confirmation of absence of PD	Not applicable
**NPD–TD**	25	38.8 ± 5.0	68 months	Retrospective confirmation of absence of PD	Not applicable

**Table 2:** Demographic and clinical features of ASD and TD children belonging to PD and NPD groups.

Group	N Children	Age (months)*	Ratio M/F	ADOS_CSS	DQ	IQ
**PD–ASD**	9	49 ± 36	8/1	6.2 ± 1	76 ± 27	91 ± 20
**PD–TD**	9	65 ± 19	5/4			
**NPD–ASD**	16	52.9 ± 11.3	13/3	4.6 ± 1.5	70 ± 20	
**NPD–TD**	25	60.5 ± 12.5	20/5			

*corresponding both to blood sampling and clinical evaluation.

### 2.2. Ethics statements

All adult participants provided written informed consent; in case of minors, parents provided written informed consent. The study protocol has been approved by the Ethical Committee of the Rome Tor Vergata University Hospital (project code: #77/13 – May 2013 and project code:#145/20 – July 2020) and it has been conducted according to the principles expressed in the Declaration of Helsinki.

### 2.3. Clinical evaluation

#### 2.3.1. Women.

Women belonging to the PD group, were categorized as affected by PD during pregnancy, on the basis of the clinical evaluation, the Diagnostic and Statistical Manual of Mental Disorders—Fifth Edition (DSM-5) [3] criteria and the score obtained at the Edinburgh Perinatal Depression Scale (EPDS) [28]. The EPDS is a self-report 10-item questionnaire which assesses depressed mood, anhedonia, anxiety, and self-harm over the past seven days using a 4 point Likert-scale; for this study we adopted 12 as cut-off for perinatal depression: < 12 Not Perinatal Depression; ≥ 12 Perinatal Depression. Moreover, the PD group (women who suffered from depression with peripartum onset), underwent, in the context of their child evaluation and blood sampling (see next paragraph), at mean distance of 47 months from pregnancy (thus from the condition of perinatal depression), to a second evaluation of depressive symptoms through the administration of the Hamilton-D scale [29,30]. The Ham-D is a 21 items clinical interview investigating depressed mood (including suicide, insomnia, agitation, anxiety, somatic and gastrointestinal symptoms, changes in weight, work and interests) over the past seven days using a 5 point Likert-scale; the HAM-D provides several score ranges of severity: ≤ 7 absence of depressed mood; 8–17 mild depression; 18–24 moderate; ≥ 25 severe. Otherwise, mothers included into the NPD group were not screened during pregnancy for the presence of mood disorders, but the absence of PD was an information retrospectively collected from their medical history during the child evaluation. More in details, clinicians performed with the women, an in-depth clinical interview investigating the prenatal, and postnatal period including psychological and psychiatric conditions.

#### 2.3.2. Children.

Children were stratified according to the maternal condition of PD during pregnancy (presence or absence of PD) and the diagnosis of ASD (presence or absence of ASD). ASD children belonging to PD group and NPD, underwent an assessment of developmental or cognitive level, autism symptoms according to the administration of the ADOS (Autism Diagnostic Observation Schedule) in the context of previous cited projects.

### 2.4. Blood sampling, RNA extraction and RT-real time PCR

Blood samples were collected from both mothers and children as part of the child assessment. Therefore, maternal blood sampling was not performed during pregnancy – concurrently with the maternal psychological condition, either PD or NPD – but, later, at an average of 47 months after delivery for the PD group, and 68 months for NPD group.

Peripheral blood mononuclear cells (PBMCs) from heparinized blood samples of all the individuals enrolled in the study were isolated by density gradient centrifugation (Lympholyte-H, Merck Darmstadt, Germany) and collected immediately after the separation. Pellets were frozen rapidly in liquid nitrogen and stored at −80°C until use. Total RNA was extracted from 0.8 × 10^5^ PBMCs using NucleoSpin RNA kit according to the manufacturer’s instructions (Macherey-Nagel, Dueren, Germany). Two hundred nanogram of DNase-treated RNA were reverse-transcribed into cDNA using Improm-II Reverse Transcription System (Promega, Fitchburg, WI, USA) according to the manufacturer’s protocol, in a total volume of 20 µl. The transcriptional levels of several HERVs, syncytins and cytokines were assessed by Real-time PCR in the Bio-Rad instrument CFX96 Real-Time System, using SYBR Green chemistry (iTaq Universal SYBR green Supermix, Biorad). Specific pairs of primers for env of HERV-H, HERV-K, HEMO, pHERV-W, syncytins and cytokines expression including IL-1β, IL-6, IL-10, TNF-α, IFN-γ were used [23,24,31–33]. To set up the Real-Time PCR a serial dilution (10-fold) was done to calculate efficiencies and correlation coefficient. The amplification efficiency was calculated by the formula [efficiency = 10^((−1)/slope) and all the primer pairs showed an efficiency ranging from 0.95 to 0.97. To verify the specificity of the primers and to exclude any false positives, DNA sequencing of PCR samples from individuals belonging to ASD and control families, was performed. Real-Time PCR included 0.20 µl of cDNA, 10 µl of SYBR green Supermix, and specific primers ranging from 100 to 200 nM, in a total volume of 20 µl, and was conducted for 1 cycle at 95°C for 5 min and then for 45 cycles of 95◦C for 10 s and 60◦C for 15 s. Each sample was analyzed in triplicate and to check out any possible contamination, a negative control was included in each experiment. The housekeeping gene β-glucuronidase (GUSB) [24] was used to normalize the results. Each experiment was completed with a melting curve analysis to confirm the specificity of amplification and the lack of non-specific products and primer dimers. Quantification was performed using the threshold cycle (Ct) comparative method and the relative expression was calculated as follows: ${\text{2}}^{-[DeltaCt(sample)-DeltaCt(calibrator)]}$, where DeltaCt (sample) = [Ct (target gene) – Ct (GUSB)], and DeltaCt (calibrator) was the mean of ΔCT of NPD mothers of TD children (for all groups of mothers) and TD children of NPD mothers (for the groups of children).

### 2.5. Statistical analysis

Statistical analysis of group-wise expression levels was performed through a non-parametric Mann–Whitney test to compare the transcriptional levels of HERVs and inflammatory mediators. Pairwise associations between HERVs and inflammatory mediators versus clinical parameters were tested through the Spearman correlation coefficient. To explore global patterns in the molecular data, we performed principal component analyses (PCA) using all quantified markers (HERV-H, HERV-K, pHERV-W, HEMO, Syn1, Syn2, IL-1β, IL-6, IL-10, TNF-α, IFN-γ), separately for the maternal and child datasets. Statistically significant comparisons were considered at p < 0.05. Data analysis was performed by using the SPSS statistical software system (version 24.0 for Windows, IBM Corp., Armonk, NY, USA).

## 3. Results

### 3.1. Demographic and clinical characteristics of the individuals included in the study

The sample was constituted by 57 mothers and 59 children and the mother-child pairs were categorized on the basis of two clinical issues: the presence or absence of maternal history of PD (PD or NPD) and the presence or absence of ASD diagnosis in offspring (ASD or TD) (see Table 1 and 2 for details). Therefore, the participants were grouped in: 16 women with history of perinatal depression (PD group: mean age 41 years ± 5.3 SD; HAM-D mean score 10.5 ± 6.4) and their respective offspring (18 children, including 2 pairs of siblings, mean age 46.7 months ± 30.5 SD), 9 with ASD (PD-ASD, mean age months 49 ± 36 SD), 9 without ASD (PD-TD, mean age months 65 ± 19 SD); 16 women without history of perinatal depression (NPD group, mean age 39.1 years ± 3.6 SD) with respective 16 offspring affected by ASD (NPD-ASD; mean age 52.9 months ± 11.3 SD); 25 mothers without perinatal depression (NPD group, mean age 38.8 years ± 5.0 SD) and their 25 children without ASD (NPD-TD: mean age 60.5 months ± 12.5 SD). Main clinical features of ASD children belonging to both groups (PD and NPD), are summarized in **Table 1**.

The Table summarizes the main demographic and clinical characteristics of mothers in the Perinatal Depression (PD) and no Perinatal Depression (NPD) groups, stratified by their child’s diagnosis (ASD or TD). Maternal age and months from delivery are shown as mean ± SD. EPDS scores refer to the screening during pregnancy in the PD cohort, while NPD mothers were retrospectively confirmed as not having experienced perinatal depression. HAM-D scores were collected at follow-up for PD mothers.

The Table reported the main demographic and results of the clinical assessment performed for ASD children of Perinatal Depression (PD) and no Perinatal Depression (NPD) group in the context of previous studies. ADOS Calibrated Severity Score (CSS) was calculated for both ADOS Edition (ADOS-2 for the ASD-PD participants; ADOS-G for the ASD-NPD group); Intellectual Quotient (IQ) is provided only for 4 ASD children of the PD-ASD group; Developmental Quotient (DQ) is provided for all the NPD-ASD group and for 5 PD-ASD children.

### 3.2. Analysis of the expression of HERVs in women who experienced perinatal depression and their offspring, stratified by ASD diagnosis

The expression of HERV-H, HERV-K, pHERV-W, HEMO and syncytins was evaluated in PBMCs obtained from PD and NPD mothers, stratified on the basis of typical development or occurrence of ASD in their children, by quantitative RT-Real time PCR analysis. The calibrator used for the relative expression analysis was the mean of ΔCT of NPD mothers of TD children (for all groups of mothers) and TD children of NPD mothers (for the groups of children).

**Fig 1:**
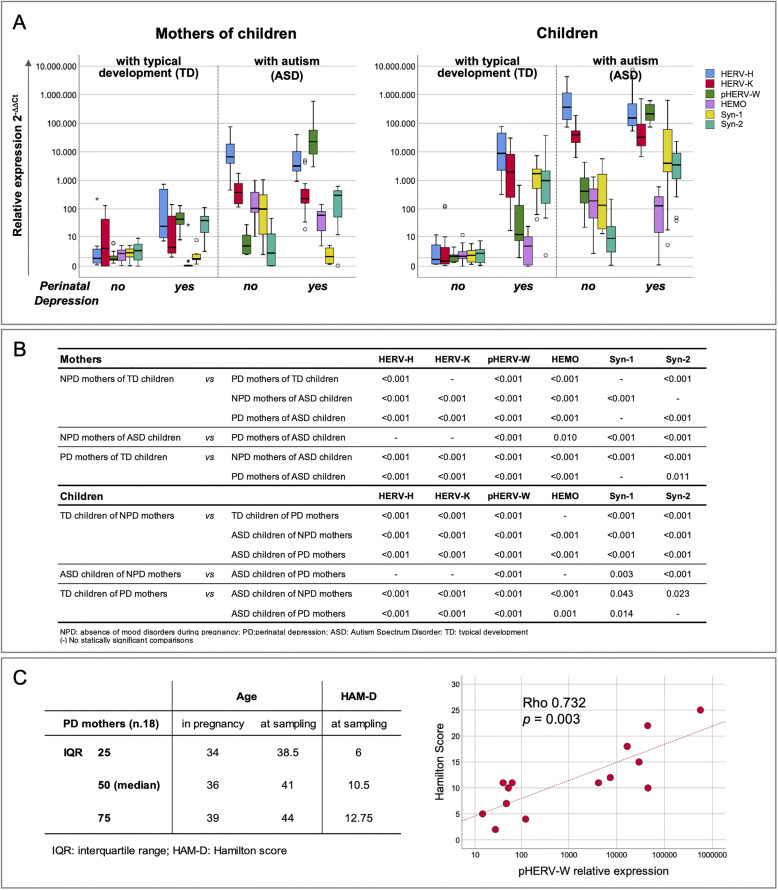
(A) Expression of HERVs, HEMO and syncytins in mothers stratified according to perinatal depression (PD) and in their typical developing and ASD children. (B) *p* values for group-wise differences examined by the nonparametric Mann–Whitney U test. (C) Scatter plots of pHERV-W expression and Hamilton-D score (HAM-D) in mothers with history of PD, who performed a second evaluation of depressive symptoms, in the context of blood sampling, at mean distance of 47 months from pregnancy.

The data are represented as box plots in **Fig 1**, panel A, *p*-values by Mann Whitney U-test are reported in **Fig 1**, panel B, and median values and interquartile range are reported in S1 Table.

Among mothers of TD children, PD women showed higher levels of expression of HERV-H, pHERV-W and Syncytin-2 (Syn-2) compared to NPD ones (*p-values* < 0.001), while HEMO was higher in NPD women (*p* < 0.001) (see S1 Table for median values and interquartile ranges).

In the group of mothers of ASD children, higher levels of HEMO and Syn-1 and lower of pHERV-W and Syn-2 were found in NPD women in comparison to PD ones (*p-values* < 0.001). When comparing mothers of ASD children and those of TD children, the highest levels of expression were found in ASD mothers, regardless of whether they had suffered from PD. Specifically, all mother of ASD children showed higher levels of HERV-H, HERV-K, pHERV-W, HEMO and syncytins expression compared to NPD mothers of TD children (*p-values* < 0.001 for all comparisons), with the exception of Syn-1 in the comparison between PD mothers of ASD children and NPD mothers of TD children and Syn-2 in the comparison between NPD mothers of ASD children and NPD mothers of TD children (see Fig 1B for details). The expression trends across markers showed comparable patterns between PD mothers of ASD children and PD mothers of TD children (*p-values* <0.001) (expect for Syn-1 levels), as well as in the comparison between NPD mothers of ASD children and PD mothers of TD children (*p-values* < 0.001). Moreover, the PD women underwent at the time of sampling (at a mean distance of 47 months from pregnancy, thus from the condition of perinatal depression) the evaluation of depression severity, through the administration of the Hamilton-D scale (HAM-D median: 10.5, IQR:6–12.75) and a positive correlation between pHERV-W and HAM-D (Rho = 0.732, *p* = 0.003) was found (Fig 1**, panel C**).

The analysis of the same genes was performed in PBMCs from TD and ASD children, stratified according to the PD of their mothers, and results are reported in Fig 1, **panel A**, *p*-values by Mann Whitney U-test are reported in Fig 1, **panel B** (see S1 Table for median values and interquartile ranges). Comparing the two groups of neurotypicals (Fig 1, **panel A**), those whose mothers had experienced PD, showed higher levels of HERV-H, HERV-K, pHERW-W and syncytins than those with NPD mothers (*p-values* < 0.001). A more similar expression profile was found comparing the two groups of ASD children since they statistically differed in pHERV-W and syncytins expression levels, which were higher in ASD children of PD mothers (*p-values* ≤ 0.003). ASD groups, regardless of PD in their mothers, showed higher levels of HERV-H, HERV-K, pHERV-W, HEMO, and syncytins compared to TD children of NPD mothers (*p-values* < 0.001 for all comparisonsand higher levels of HERV-H, HERV-K, pHERV-W, HEMO, and Syn-1 compared to those of PD mothers (*p-values* ≤ 0.043). Higher levels of Syn-2 were found in TD and ASD children of PD mothers, compared to the other two groups (*p* ≤ 0.01, see Fig 1, **panel B** for details). No correlations were found between HERV expression levels and clinical parameters in all children’s groups.

### 3.3. Analysis of the expression of inflammatory mediators in women who experienced perinatal depression and their offspring, stratified by ASD diagnosis

**Fig 2:**
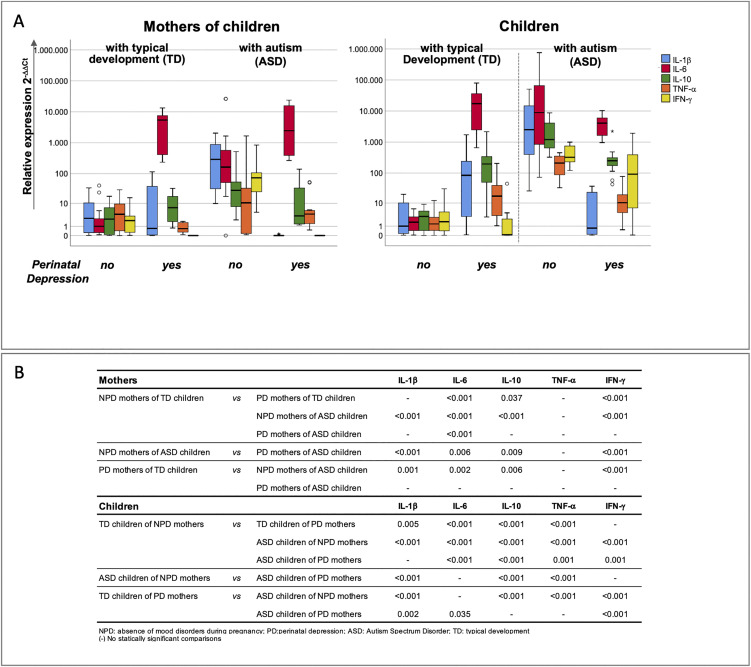
(A) Expression of IL-1β, IL-6, IL-10, TNF-α and IFN-γ in mothers stratified according to perinatal depression and their typical developing and ASD children. (B) *p* values for group-wise differences examined by the nonparametric Mann–Whitney U test.

The expression of IL-1β, IL-6, IL-10, TNF-α and IFN-γwas evaluated in PBMCs from all mothers stratified on the basis of typical development or the occurrence of ASD in their children (Fig 2, **panel A**), *p*-values by Mann Whitney U-test are reported in Fig 2, **panel B**, and median values and interquartile range are reported in S1 Table.

Comparing the two groups of mothers of TD children (Fig 2, **panel A**) (see S1 Table for median values and interquartile ranges), PD women showed higher levels of expression of IL-6 and IL-10 than NPD mothers (*p-values* ≤ 0.037), and lower of IFN-γ (*p* < 0.001).

Among mothers of ASD children, PD women showed higher levels of IL-6 compared with NPD ones (*p* = 0.006) and lower levels of IL-1β,IL-10 and IFN-γ(*p-values* ≤ 0.009). In the comparison with NPD mothers of TD children, all mothers of ASD children, regardless of perinatal depression, showed higher levels of expression of IL-6 (*p-values* < 0.001), while IL-1β, IL-10, and IFN-γ expression levels were higher only NPD mothers of ASD children (*p* < 0.001). With respect to PD women with TD children, NPD mothers of ASD children exhibited higher levels of IL-1β, IL-10, TNF-αand IFN-γ (*p-values* ≤ 0.006) and lower levels of IL-6 (*p* = 0.002). No statically significant changes were found in the comparison between the two groups of PD mothers.

The expression of the same inflammatory mediators was evaluated in PBMCs from TD and ASD children stratified according to PD in mothers, by quantitative RT-Real time PCR analysis. The data are represented as box plots in Fig 2, **panel A**; *p*-values by Mann Whitney U-test are reported in Fig 2, **panel B**. Among neurotypicals (Fig 2, **panel A**) (see S1 Table for median values and interquartile ranges), those with PD mothers showed higher levels of expression of IL-1β, IL-6, IL-10 and TNF-α than children of NPD mothers (*p-values* ≤ 0.005). Among ASD children, those with NPD mothers showed higher levels of IL-1β, IL-10 and TNF-α compared with children with PD ones (*p-values* ≤ 0.05). All ASD children, regardless PD in their mothers, showed higher levels of expression of IL-6, IL-10, TNF-α and IFN-γ compared to TD individuals of NPD mothers (*p-values* ≤ 0.001) while IL-1β levels were higher only in ASD children of NPD mothers (*p* < 0.001). In the comparison with TD children of PD mothers, ASD children with NPD mothers exhibited higher levels of IL-1β, IL-10, TNF-α and IFN-γ (*p-values* < 0.001) while ASD children of PD mothers exhibited lower levels of IL-1β, IL-6, and IFN-γ (*p-values* ≤ 0.035). No correlations were found between cytokines expression levels and clinical parameters in all children’s groups.

Exploratory PCA of the molecular markers did not reveal clear clustering of samples. In mothers, PC1 and PC2 explained 36.8% and 27.7% of the variance, respectively, with PD and NPD subjects largely intermingled in the PC1–PC2 space. In children, PC1 and PC2 explained 89.5% and 5.5% of the variance, respectively, with ASD and TD samples also showing no evident separation. The PCA plots are provided in S1 Fig, in which the first two principal components (PC1–PC2) were used for visualisation, with subjects colour-coded according to PD vs NPD (mothers) and ASD vs TD (children) (S1 Fig).

## 4. Discussion

In the present study we showed that mothers of children with ASD exhibit increased expression of HERV-H and HEMO compared with mothers of typically developing children. These findings are consistent with our previous studies reporting abnormal HERV activation both in autistic children and their mothers [23,24], reinforcing the hypothesis of a shared mother-child molecular profile, potentially involved in the etiopathogenesis of autism. Interestingly, we also show here that mothers who had suffered from perinatal depression displayed even higher levels of HERV-H and HEMO than those without history of perinatal depression.

Several HERVs are known to play important roles in mammalian development and differentiation [20,34–37]. Syncytin-1 and syncytin-2, encoded by the HERV-W and HERV-FRD families, respectively, possess fusogenic properties that are crucial for trophoblast fusion and placental development. These envelope proteins also contribute to the establishment of maternal–fetal immune tolerance [38,39]. HERV-K expression also increases during embryogenesis in parallel with the embryonic transcription factor OCT4 and seems to be involved in the immune protection of human embryos against viruses sensitive to the interferon-induced transmembrane protein 1 [40]. In addition, the well-preserved human endogenous MER34 ORF (HEMO) is highly expressed in embryos at different phases of development, and in the placenta during pregnancy, and released in the blood of pregnant women [33]. Therefore, due to their crucial role in pregnancy and fetal development, altered activity of HERVs could impact neurodevelopment. Concerning the cytokine expression, PD mothers, regardless the occurrence of autism in their children, showed higher levels of IL-6 and IL-10 with respect to NPD mothers with neurotypical children. Interestingly, higher levels of IL-1β, IL-10 and IFN-γ were found in PD mothers of ASD children compared to NPD mothers of ASD children. The simultaneous increase of inflammatory cytokines and IL-10, known as an anti-inflammatory mediator, could be related to an attempt to regulate/restore the inflammatory response with the aim of limiting this vicious cycle in which HERVs stimulated the immune system and the products of the immune system are in turn able to support the activation of HERVs [24,25]. These observations are in line with substantial evidence indicating dysregulated immune activity in perinatal depression. As such, IL-1β, IL-6 and TNF-α levels were positively associated with perinatal depressive symptoms during pregnancy, though some studies showed no association [41,42]. For IFN-γ, any association between prenatal levels and depression emerged, while for IL-10, it has been demonstrated an upregulation in women during the first trimester with postpartum onset of depression but not in those who were already depressed prior to pregnancy [42,43]. It is known that under physiological conditions, most HERVs are silenced mainly through epigenetic mechanisms [44,45]. However, their transcription can be reactivated by a wide range of environmental stimuli, including ultraviolet radiation, nutrients, drugs, psychological stress, and infectious agents such as viruses, protozoa and components of the gut microbiota. Cytokines and hormonal factors can also modulate HERV expression, reflecting the intrinsic responsiveness of these elements to immunological and endocrine cues [46]. Among exogenous triggers, viral infections exert strong effects on HERV activation. Several viruses, including HIV, HTLV-1, EBV, and HHV-6, can directly induce HERV transcription, partly by reducing epigenetic repression in a cell-intrinsic fashion [47–49]. The immune response to exogenous viruses may also be compromised by similarities between HERV-derived epitopes and retroviral antigens, while HERV sequences themselves may serve as substrates for recombination with exogenous retroviruses, further promoting activation [48]. *Mycobacterium avium* subspecies *paratuberculosis* (MAP), a pluripotent driver of human disease comprising type one diabetes (T1D), multiple sclerosis (MS), rheumatoid arthritis, autoimmune thyroiditis and other disorders, has been shown to activate HERVs. This activation has been documented in T1D [50], MS [51,52], non-solar uveal melanoma [53], colon cancer [54], glioblastoma [55], and amyotrophic lateral sclerosis [56], suggesting that HERV responsiveness to microbial stimuli may be relevant across multiple disease contexts. Once activated, HERVs can produce mRNA, viral particles, and viral proteins, all of which may contribute to disease onset or accelerate disease progression [21,46]. These HERV-derived products can be recognized by pattern-recognition receptors, eliciting innate immune responses and leading to the release of inflammatory cytokines and chemokines [57–59]. This mutual interaction supports the concept of a vicious cycle, in which HERV-induced inflammation further enhances HERV activation. Recent evidence also indicates that the SARS-CoV-2 spike protein can induce HERV-W ENV expression both in PBMCs from healthy donors and in cancer cell lines, preceding the induction of IL-6 [31,60]. This observation further links HERV activation to dysregulated immune responses in viral infections [61]. As a consequence, inflammatory mediators induced by HERVs could, in turn, further increase HERV activity [62,63]. Indeed, NF-kB-associated transcription factors can bind the LTR of HERV-K and the interferon (IFN)-stimulated regulatory element, leading to the production of pro-inflammatory cytokines, including type I IFNs [62,64,65]. Accordingly, the expression of HERV-H, HERV-K, and HERV-W, at mRNA level, was increased by TNF-α, driving the translocation of NF-kB into the nucleus [64]. Of note, the associations between the expression of HERVs and cytokines observed in this study do not allow causal inferences regarding the relationship between HERV activity, inflammation, and neurodevelopmental outcomes. However, existing literature show that specific HERV families can modulate immune pathways and participate in neuroinflammatory processes [49,57,58], as suggested by an *in vitro* approach, demonstrating that the induction of HERV-W ENV expression by the SARS-CoV-2 spike protein occurs prior to the expression of IL-6 in PBMCs from healthy donors [31,60]. In the present study, we found that pHERV-W was expressed in both groups of PD women, and the highest levels were found in those with ASD children. In addition, in the comparison with the group of NPD mothers of ASD children, higher levels of pHERV-W as well as of syncytin-2 were found in PD women, regardless of the occurrence of autism in their children. Several studies have suggested potential associations between HERVs and neuropsychiatric disorders as schizophrenia and bipolar disorder in which HERV-W expression was associated with increased serum levels of inflammatory cytokines and higher childhood maltreatment scores [66–68]. However, the precise mechanisms through which HERVs might contribute are not yet fully understood. Altered HERV expression has been demonstrated also in post-mortem brain tissues from patients suffering from major depression, as the reduction of HERV-W GAG protein has been detected in the cingulate gyrus and hippocampus, indicating a potential role in such pathophysiological circuitry [26]. In contrast, in the present study we observed high ENV transcriptional levels of HERV-W in PBMCs from PD women. This different trend could be due to the different gene, tissue and product considered. Therefore, further studies are needed to investigate the functions of the different products (pol, gag, env etc.) of HERVs under both physiological and pathological conditions.

In the present study we also showed a general deregulation of HERVs, syncytins and cytokines in both neurotypical and ASD children of PD mothers with respect to neurotypical children born to NPD mothers. Interestingly, when comparing groups of children of PD mothers, higher levels of HERVs, Syn-1, IL-1β, IL-10 and TNF-α were found in ASD children. Of note, the study includes two cohorts recruited in different years and under distinct study protocols, which may have introduced variability related to sample handling or environmental factors across recruitment periods. Of note, the impact of the exposure to SARS-CoV-2 during the COVID-19 pandemic represents a non-negligible environmental stressor that has undoubtedly had an impact on the gene expression profile identified in the study cohort. In addition, this recruitment gap is one of the major limitations linked to comparability of the PD and NPD groups. Future larger cohort studies will allow us to verify these aspects in a robust manner.

The interplay between HERV activity and inflammatory pathways suggests that HERVs might participate in neuroinflammatory mechanisms potentially relevant to mood regulation and depressive symptoms. Brain inflammation is thought to play a role in some cases of depression, although no definitive proof has been found. The activation of HERV elements may trigger immune responses and contribute to neuroinflammation, which could affect mood-regulating neurotransmitters and synaptic plasticity. This hypothesis is in line with our previous results and, the common molecular profile between mother and children could likely be the result of influences coming from the shared environment, maternal status, genetic predisposition, or postnatal factors. Consistently, we have already shown that ASD children and their mothers shared the altered expression of several HERVs and cytokines, a molecular profile absent in fathers, suggesting a mother-child association within the ASD families [24]. This association is corroborated from preclinical findings. Specifically, in the VPA mouse model of autism, we previously observed altered endogenous retroviruses (ERVs) and cytokine expression in embryos and in postnatal blood and brain samples. Notably, in VPA-exposed mice, both ERV upregulation and behavioral abnormalities were transmitted across generations via the maternal lineage, supporting a key role of maternal ERV dysregulation in shaping neurodevelopmental outcomes [69]. We also reported dysregulation of selected ERVs and inflammatory mediators in maternal decidua and extra-embryonic tissues of the idiopathic BTBR T + tf/J model, indicating that ERV-related alterations emerge already during intrauterine life and are closely linked to the maternal compartment [70]. Relevant with this preclinical evidence, our results in humans show that ASD children of PD mothers display higher levels of pHERV-W and syncytins compared with ASD children of NPD mothers. Finally, our data showed that PD in mothers is associated with altered expression of HERVs in both neurotypical and ASD children. However, maternal depression alone may not fully explain the elevated HERV expression detected in ASD children; additional environmental or genetic factors are likely required, consistent with a multi-hit model of pathogenesis [71].

Overall, these findings appear to strengthen the view that maternal depression, HERV dysregulation and immune alteration converge in shaping both maternal and child molecular profiles, potentially contributing to the risk of neurodevelopmental disorder. However, the present pilot study is limited by the small sample size, the retrospective assessment of perinatal depression in the NPD group, the different timing of clinical evaluations, and the long time between pregnancy and blood sampling. Although these limitations must be considered, the findings still provide an initial basis for future studies conducted during pregnancy, where clinical and molecular assessments can be integrated. We also acknowledge that postnatal environmental exposures, life events, and possible subsequent depressive episodes may have influenced the molecular profiles measured years after pregnancy. These factors cannot be separated from the effects of perinatal depression in the present design and therefore represent an additional limitation of this work.

In our samples, a positive correlation emerged between pHERV-W expression and the severity of depressive symptoms in PD mothers, suggesting that HERV activity may reflect current affective states. Although preliminary, this observation is in line with evidence that HERV expression may vary according to clinical trajectories in psychiatric conditions. Further research is needed to clarify whether specific HERV signatures could serve as clinically informative markers. This hypothesis is supported by findings from ADHD patients, where HERV-H levels decrease in patients improving under methylphenidate, with molecular changes preceding clinical improvement [72].

Taken together, our data indicates that PD associated with the altered activity of HERVs and cytokine expression, with a more pronounced dysregulation in ASD offspring, could be considered an additional risk factor in ASD pathogenesis. Nevertheless, the contribution of HERVs in such scenario must be verified to reach solid conclusions. Consistent with this interpretation, exploratory PCA did not reveal clear clustering by clinical status. This observation represents a further limitation of the present study and may be due to the sample being limited and clinically heterogeneous to the extent that the differences in individual markers that are statistically significant do not translate into distinct, separable molecular profiles. Maternal depression alone, however, is unlikely to fully account for the elevated HERV expression observed in ASD children; additional environmental or genetic contributors are likely involved, consistent with multi-hit models of neurodevelopmental risk. Future prospective studies, ideally involving biological sampling during pregnancy and early infancy, will be essential to clarify these interactions and to determine whether maternal perinatal depression contributes to increased vulnerability to autistic traits in offspring.

## 5. Conclusions

The rising prevalence of maternal perinatal depression underscores the need to investigate its biological effects and clinical impact on maternal and child health. The characterization of the expression of HERVs and cytokines in women with perinatal depression and in their ASD children highlights elements that may reflect a common mechanism increasing the risk of neurodevelopmental disorders in offspring. Evidence from both pre-clinical models and human studies supports the role of dysregulated ERV expression in autism as a contributing factor in creating an adverse environment for neurodevelopment [69,73–76]. Perinatal depression being associated with altered HERV activity, may act as an additional contributing factor in the multifactorial pathogenesis of autism, although a causal relationship has not been demonstrated and, many children born to mothers with perinatal depression do not develop autism. The findings of the present study do not provide evidence of a direct mechanistic link between PD, HERV dysregulation and ASD; rather, they should considered as preliminary, hypothesis-generating evidence.

Within this framework and in line with multi-factorial models of disease vulnerability, it can be assumed that exposure to an unfavorable environment during fetal development may trigger HERV expression, which in turn, may sustain a chronic inflammatory status, likely via epigenetic mechanisms [45,71,77,78], potentially influencing neurodevelopment.

## Supporting information

S1 Fig Principal Component Analysis (PCA). PCA and hierarchical clustering of gene expression of all quantified markers, separately for the maternal and child datasets.
(XLSX)

S1 Table Median value and interquartile range (IQR) of the expression levels of HERVs and inflammatory mediators in Perinatal Depression (PD) and no Perinatal Depression (NPD) mothers with corresponding Autism Spectrum Disorder (ASD) and Typical Development (TD) children. (XLSX)
